# *H*_∞_ Observer Based on Descriptor Systems Applied to Estimate the State of Charge

**DOI:** 10.3390/e24030420

**Published:** 2022-03-17

**Authors:** Shengya Meng, Shihong Li, Heng Chi, Fanwei Meng, Aiping Pang

**Affiliations:** 1 School of Control Engineering, Northeastern University at Qinhuangdao, Qinhuangdao 066004, China; 2001944@stu.neu.edu.cn (S.M.); 2071918@stu.neu.edu.cn (H.C.); mengfanwei@neuq.edu.cn (F.M.); 2 School of Automation, Aviation University Air Force, Changchun 130000, China; 3 College of Electrical Engineering, Guizhou University, Guiyang 550025, China; appang@gzu.edu.cn

**Keywords:** descriptor systems, SOC estimation, *H*_∞_ observer, disturbance suppression performance

## Abstract

This paper proposes an $H_{\infty }$ observer based on descriptor systems to estimate the state of charge (SOC). The battery’s open-current voltage is chosen as a generalized state variable, thereby avoiding the artificial derivative calculation of the algebraic equation for the SOC. Furthermore, the observer’s dynamic performance is saved. To decrease the impacts of the uncertain noise and parameter perturbations, nonlinear $H_{\infty }$ theory is implemented to design the observer. The sufficient conditions for the $H_{\infty }$ observer to guarantee the disturbance suppression performance index are given and proved by the Lyapunov stability theory. This paper systematically gives the design steps of battery SOC $H_{\infty }$ observers. The simulation results highlight the accuracy, transient performance, and robustness of the presented method.

## 1. Introduction

Over the past few years, renewable energy vehicles (REVs) have become a mainstream consumer option, so related research about REV batteries has been of great interest [1]. The state of charge (SOC) is a percentage of the remaining capacity to the actual capacity of the battery, which is a vital indicator to evaluate battery performance [2,3]. Accurately tracking the SOC can dramatically avoid battery overcharge or overdischarge, thereby extending the battery life. However, due to a series of complex electrochemical reactions inside the battery, it is often impossible to obtain the SOC directly through the sensors. In other words, SOC can only be estimated by the measurable electrical signals and battery parameters. Even worse, battery parameters are affected by external factors such as temperature, battery age, and noise in electrical signals [4]. Accordingly, the SOC observer needs to provide sufficient estimation accuracy even in noise and parameter perturbations, which is a daunting task.

**Figure 1:**
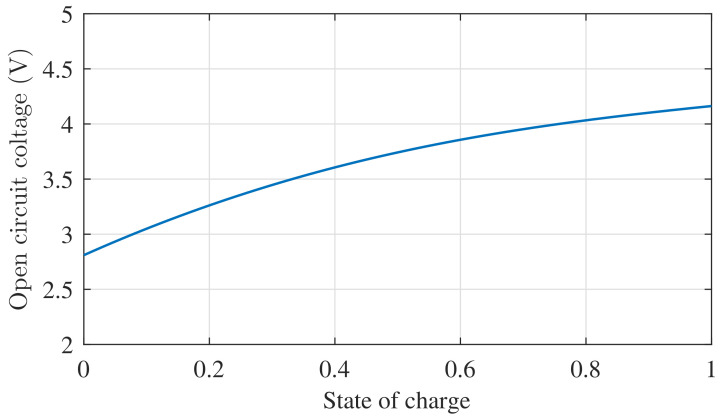
The relationship between SOC and OCV.

A variety of algorithms are proposed to estimate SOC, such as the coulomb counting method (CCM), open-circuit voltage method (OCVM), Kalaman filter (KF), sliding-mode observer (SMO), $H_{\infty }$ observer, neural network algorithm, proportional-integral (PI) observer, and adaptive observer [5,6,7]. The CCM estimates SOC by continuously measuring and integrating the current in time. The main drawbacks of CCM are two-fold: the first is that CCM highly depends on the initial value of observers, and the second is that it is known as an open-loop method whose estimation value will drift in the long term [8]. Alternatively, because of the one-to-one correspondence (as shown in Figure 1) between the SOC and the open-circuit voltage (OCV), the OCVM estimates the SOC by measuring the OCV of the battery without load. However, this technique fails to estimate SOC online. Due to their drawbacks, CCM and OCVM are never utilized separately in practical applications [9]. The KF and SMO are widely employed in the field of SOC estimation [10,11]. Nevertheless, due to the assumption of a noise signal Gaussian, the KF falls short when the system has noise or unmodeled dynamics [12,13]. The SMO is commonly used for SOC estimation due to its robustness. In [14], an OCV–SOC formula was modeled by the Nernst equation, and a SMO was proposed to estimate SOC; simulation results validate its accuracy. However, the estimate error of SOC may fluctuate because of the discontinuous input. A new SMO, based on the two-circuit model presented in [15], exhibits good performance. However, without accurate initial states, the SMO in [15] takes longer to track the true SOC.

The influence of possible error sources on the SOC observation was analyzed in [16], and the results show that measurement noise and modeling errors are the main factors that limit the observation accuracy. The $H_{\infty }$ observer is a promising tool to handle unknown noise and modeling errors, and its effectiveness under a variety of operating conditions has been confirmed by experiments [17,18,19,20]. Based on the OCV–SOC formula, an $H_{\infty }$-switched observer was presented in [21]; the experimental results confirm that, compared with the KF, both the accuracy and robustness of SOC estimation are improved by its use.

Regardless of the above approaches, it is impossible to ignore the piecewise nonlinear function of OCV versus SOC shown in Figure 1. In the battery model, the SOC fails to be expressed explicitly in the state equation, which brings difficulties to the design observer. In [22,23], the piecewise nonlinear function was linearized and differentiated before the observer design. However, the differential operation produced two problems:

1. The derivation of the piecewise function increased the order of the observer, which did not match the original system, and the observer error was not converged potentially;
2. The derivation of the current was ignored completely, so the dynamic performance of the observer became worse.

There are both differential equations and algebraic equations in battery systems. Such systems are also called descriptor systems, singular systems, or differential-algebraic systems [24,25]. To avoid the differentiation of the OCV–SOC formula, it is feasible to design the observer after modeling the battery as a descriptor system. Various methods are developed to design observers for descriptor systems [26].

The main objective of this paper is to design a noncomplex observer to estimate SOC accurately. To balance accuracy and complexity, this paper innovatively models the battery as a descriptor system. The $H_{\infty }$ theory is applied to design the observer to improve disturbance suppression performance. Compared with the traditional SOC estimation method, the method proposed in this paper can accurately estimate the SOC online, and does not require an accurate initial value. The designed observer exhibits good robustness in the presence of noise.

This paper is organized as follows. In Section 2, for the equivalent circuit model, a descriptor system with state variable OCV is established. In Section 3, the $H_{\infty }$ observer is proposed. The sufficient conditions to solve the observer are given and proved. In Section 4, several simulation experiments verify the accuracy and robustness of the proposed method. Section 5 summarizes the contribution of this paper.

Notations: $M^{+}$ is the generalized inverse of matrix *M*, satisfying $MM^{+}M=M$. *I* denotes an identity matrix with appropriate dimensions. 0 is the zero matrix with appropriate dimensions.

## 2. Battery Model

**Figure 2:**
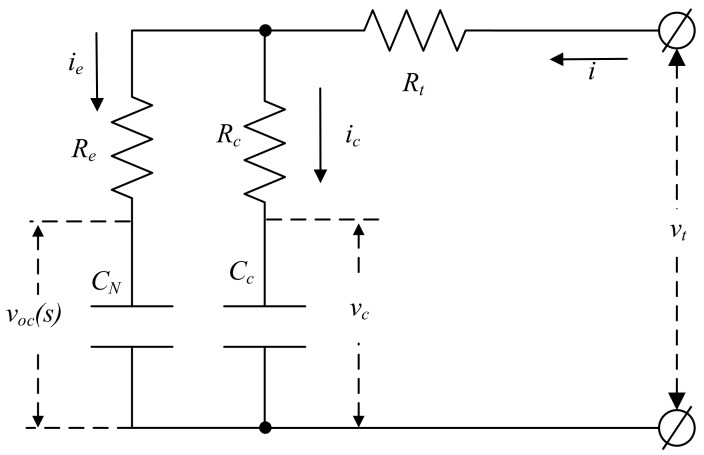
RC equivalent circuit model of the battery.

A resistance–capacitance (RC) equivalent circuit model is used to build a dynamic model of the battery, as shown in Figure 2, where the variable *s* represents the SOC. $C_{N}$ is the nominal capacity of the battery. $v_{oc}$ represents the OCV, which is the function of SOC. $v_{c}$ is the voltage across the polarized capacitor $C_{c}$. $R_{e}$ and $R_{c}$ represent the conduction resistance and the diffusion resistance, respectively; $i_{e}$ and $i_{c}$ are the currents of the two branches; $R_{t}$ is the terminal resistance; $v_{t}$ is the measurable terminal voltage, and *i* is the charge and discharge current.

From the definition of SOC, the dynamic relationship of SOC is $\dot{s}=\frac{i_{e}}{C_{N}}$. In addition, the aforementioned nonlinear function $v_{oc}$ can be reasonably approximated as $v_{oc}=k_{1}s+k_{2}+\Delta f_{1}$, where $\Delta f_{1}$ is the nonlinearity of the OCV–SOC relationship. The two constants $k_{1}$ and $k_{2}$ can be determined by fitting the curve in Figure 1. From Kirchhoff’s law and Figure 2, the dynamic equations of the battery are:

(1) $$\begin{array}{cc} \dot{s}= & \frac{-v_{oc}+v_{c}}{C_{N}\left(R_{e}+R_{c}\right)}+\frac{iR_{c}}{C_{N}\left(R_{e}+R_{c}\right)}+\Delta f_{2},\\ {\dot{v}}_{c}= & \frac{v_{oc}-v_{c}}{C_{c}\left(R_{e}+R_{c}\right)}+\frac{iR_{e}}{C_{c}\left(R_{e}+R_{c}\right)}+\Delta f_{3},\\ v_{t}= & \frac{R_{c}v_{c}+R_{e}v_{oc}}{R_{e}+R_{c}}+\left(\frac{R_{e}R_{c}}{R_{e}+R_{c}}+R_{t}\right)i, \end{array}$$

where $\Delta f_{2}$ and $\Delta f_{3}$ are the uncertainties caused by modeling accuracy.

In this model, s∈(0,1) is the independent variable of the $v_{oc}$, which essentially introduces a piecewise algebraic constraint. To solve this piecewise algebraic system state estimation problem, Refs. [22,23] ignore the change of the current to derive the $v_{oc}$ and $v_{t}$, respectively, and model the system as a third-order system which is primordially two-order. In the above modeling process, the derivation operation increases the order of the system, and it is doubtful whether the observer error converges. In the actual application of batteries, especially in the course of REVs, the current of the battery is constantly changing. Therefore, it is obviously unreasonable to completely ignore the derivative of the current.

Motivated by these considerations, this paper regards the OCV–SOC function as an algebraic constraint between state variables, thereby modeling the system battery as a descriptor system. $x={\left[\begin{array}{ccc} s^{T} & v_{c}^{T} & v_{oc}^{T} \end{array}\right]}^{T}$ is identified as a state variable, $u={\left[\begin{array}{cc} i^{T} & 1 \end{array}\right]}^{T}$, and $\omega ={\left[\begin{array}{ccc} \Delta f_{1}^{T} & \Delta f_{2}^{T} & \Delta f_{3}^{T} \end{array}\right]}^{T}$; then, the battery is modeled as a descriptor system (2) with n=3 dimensions.

(2) $$\begin{array}{cc} E\dot{x} & =Ax+Bu+D_{1}\omega ,\\ y & =Cx+Du, \end{array}$$

where:

$$\begin{array}{cc} E & =\left[\begin{array}{ccc} 1 & 0 & 0\\ 0 & 1 & 0\\ 0 & 0 & 0 \end{array}\right],\;A=\left[\begin{array}{ccc} 0 & \frac{1}{C_{N}\left(R_{e}+R_{c}\right)} & \frac{-1}{C_{N}\left(R_{e}+R_{c}\right)}\\ 0 & \frac{-1}{C_{c}\left(R_{e}+R_{c}\right)} & \frac{1}{C_{c}\left(R_{e}+R_{c}\right)}\\ k_{1} & 0 & -1 \end{array}\right],\\ B & =\left[\begin{array}{cc} \frac{R_{c}}{C_{N}\left(R_{e}+R_{c}\right)} & 0\\ \frac{R_{e}}{C_{c}\left(R_{e}+R_{c}\right)} & 0\\ 0 & k_{2} \end{array}\right],\;C=\left[\begin{array}{ccc} 0 & \frac{R_{c}}{R_{e}+R_{c}} & \frac{R_{e}}{R_{e}+R_{c}} \end{array}\right],\\ D & =\left[\frac{R_{e}R_{c}}{R_{e}+R_{c}}+R_{t}\;0\right],\;D_{1}=\left[\begin{array}{ccc} 0 & 1 & 0\\ 0 & 0 & 1\\ 1 & 0 & 0 \end{array}\right]. \end{array}$$

Before the observer design, assume that the descriptor system (2) satisfies Assumption 1.

**Assumption** **1.**
(3) $$\mathrm{rank}\left[\begin{array}{cc} E & A\\ 0 & E\\ 0 & C \end{array}\right]=n+\mathrm{rank}E,$$

*where n is the number of state variables.*

Under Assumption 1, the descriptor system (2) is impulse observable, which guarantees there exists an observer to track the states. Actually, this assumption is not strict and easy to achieve in battery models.

## 3. $H_{\infty }$ Observer

Design an $H_{\infty }$ observer described as follows:

(4) $$\begin{array}{cc} \dot{z}= & Hz+J\bar{y}+Mu,\\ \hat{x}= & Pz-Q\Phi Bu+R\bar{y}, \end{array}$$

where $z\in R^{r}$ is the state variable of the observer, $\hat{x}\in R^{n}$ is the estimated value of the battery state, *H*, *J*, *M*, *P*, *Q*, and *R* are all unknown matrices with appropriate dimensions, and $\bar{y}=y-Du$ is the virtual output. Φ satisfies ΦE=0.

The $H_{\infty }$ observer design target can be expressed as designing a stable observer (4) to satisfy that:

1. With ω=0, the estimate error $e=x-\hat{x}$ is asymptotically stable;
2. With ω≠0, for a prescribed level of noise γ>0, ${\left\|\;e\;\right\|}_{L_{2}}<\gamma \;{\left\|\;\omega \;\right\|}_{L_{2}}$ will be satisfied.

Define the error δ=z−NEx, where *N* is of appropriate dimensions. Then, one has:

(5) $$\begin{array}{cc} \dot{\delta }= & \dot{z}-NE\dot{x}\\ = & H\delta +\left(HNE+JC-NA\right)x+\left(M-NB\right)u-ND_{1}\omega ,\\ e= & \hat{x}-x\\ = & P\delta +\left(PNE+Q\Phi A+RC-I_{n}\right)x+Q\Phi D_{1}\omega , \end{array}$$

where ΦE=0 is applied. Under Assumption 1, to make the error system (5) be a homogeneous linear differential equation for δ, the observer (4) should satisfy:

(6) $$\left[\begin{array}{ccc} H & \psi & J\\ P & Q & R \end{array}\right]\left[\begin{array}{c} N^{{}^{\prime }}E\\ \Phi A\\ C \end{array}\right]=\left[\begin{array}{c} N^{{}^{\prime }}A\\ I_{n} \end{array}\right],$$

(7) M=NB,

where ψ is an arbitrary matrix of appropriate dimension and $N^{{}^{\prime }}=N+\psi \Phi$.

To facilitate the analysis, define $\varphi_{1}=-ND_{1}$ and $\varphi_{2}=Q\Phi D_{1}$; then, the dynamics of the error system are given by:

(8) $$\begin{array}{cc} \dot{\delta } & =H\delta +\varphi_{1}\omega ,\\ e & =P\delta +\varphi_{2}\omega . \end{array}$$

Notice that Equation (6) can be solvable if and only if:

(9) $$\mathrm{rank}\left[\begin{array}{c} N^{{}^{\prime }}E\\ \Phi A\\ C \end{array}\right]=n.$$

With Equation (9), the solution of Equation (6) can be described as:

(10) $$\begin{array}{cccccc} \; & H=\Gamma_{H}+\eta_{1}\Delta_{P}, & \; & \psi =\Gamma_{\psi }+\eta_{1}\Delta_{Q}, & \; & J=\Gamma_{J}+\eta_{1}\Delta_{R},\\ \; & P=\Gamma_{P}+\eta_{2}\Delta_{P}, & \; & Q=\Gamma_{Q}+\eta_{2}\Delta_{Q}, & \; & R=\Gamma_{R}+\eta_{1}\Delta_{R},\\ \; & \varphi_{1}=\Gamma_{\varphi_{1}}+\eta_{1}\Delta_{\varphi_{1}}, & \; & \varphi_{2}=\Gamma_{\varphi_{2}}+\eta_{2}\Delta_{\varphi_{2}}, \end{array}$$

where $\eta_{1}$ and $\eta_{2}$ are of appropriate dimension. Define the following matrices:

$$\begin{array}{ccccccc} \; & \Gamma_{P}=\Omega^{+}\left[\begin{array}{c} I\\ 0\\ 0 \end{array}\right], & \; & \Delta_{P}=\left(I-\Omega \Omega^{+}\right)\left[\begin{array}{c} I\\ 0\\ 0 \end{array}\right], & \; & \Gamma_{H}=N^{{}^{\prime }}A\Gamma_{P},\\ \; & \Gamma_{Q}=\Omega^{+}\left[\begin{array}{c} 0\\ I\\ 0 \end{array}\right], & \; & \Delta_{Q}=\left(I-\Omega \Omega^{+}\right)\left[\begin{array}{c} 0\\ I\\ 0 \end{array}\right], & \; & \Gamma_{\psi }=N^{{}^{\prime }}A\Gamma_{Q},\\ \; & \Gamma_{R}=\Omega^{+}\left[\begin{array}{c} 0\\ 0\\ I \end{array}\right], & \; & \Delta_{R}=\left(I-\Omega \Omega^{+}\right)\left[\begin{array}{c} 0\\ 0\\ I \end{array}\right], & \; & \Gamma_{J}=N^{{}^{\prime }}A\Gamma_{R}, & \end{array}$$

$$\begin{array}{ccc} \; & \Gamma_{\varphi_{1}}=-N^{{}^{\prime }}D_{1}-\Gamma_{\psi }\Phi D_{1},\; & \Delta_{\varphi_{1}}=-\Delta_{Q}\Phi D_{1},\\ \; & \Gamma_{\varphi_{2}}=\Gamma_{Q}\Phi D_{1},\; & \Delta_{\varphi_{2}}=\Delta_{Q}\Phi D_{1}, \end{array}$$

where $\Omega =\left[\begin{array}{c} N^{{}^{\prime }}E\\ \Phi A\\ C \end{array}\right]$.

The following theorem gives the sufficient conditions for error system (8) to be stable and ${\left\|\;e\;\right\|}_{L_{2}}<\gamma \;{\left\|\;\omega \;\right\|}_{L_{2}}$ with (6) and (7).

**Theorem** **1.** *For a prescribed level of noise γ>0, under δ=0, the error system (8) with (6) and (7) is asymptotically stable for ω=0, and satisfies ${\left\|\;e\;\right\|}_{L_{2}}<\gamma \;{\left\|\;\omega \;\right\|}_{L_{2}}$ for ω≠0, if there exists a matrix $X=X^{T}>0$ and matrices $X_{\eta_{1}}$ and $\eta_{2}$, such that the following linear matrix inequality (LMI) is satisfied:*

(11) $$\begin{array}{cc} \Sigma = & \left[\begin{array}{ccc} \sigma_{1} & \sigma_{2} & \sigma_{3}\\ \sigma_{2}^{T} & -\gamma^{2}I & \sigma_{4}\\ \sigma_{3}^{T} & \sigma_{4}^{T} & -I \end{array}\right]<0, \end{array}$$

*where:*

$$\begin{array}{cc} \sigma_{1}= & \Gamma_{H}^{T}X+X\Gamma_{H}+\Delta_{P}^{T}X_{\eta_{1}}^{T}+X_{\eta_{1}}\Delta_{P},\;\sigma_{2}=X\Gamma_{\varphi_{1}}+X_{\eta_{1}}\Delta_{\varphi_{1}},\\ \sigma_{3} & =\Gamma_{P}+\eta_{2}\Delta_{P},\;\sigma_{4}=\Gamma_{\varphi_{2}}^{T}+\Delta_{\varphi_{2}}^{T}\eta_{2}^{T},\;X_{\eta_{1}}=X\eta_{1}. \end{array}$$

**Proof** **of Theorem 1.** According to (10) and (11), one can obtain:

(12) $$\begin{array}{c} \left[\begin{array}{ccc} H^{T}X+XH & X\varphi_{1} & P^{T}\\ \varphi_{1}^{T}X & -\gamma^{2}I & \varphi_{2}^{T}\\ P & \varphi_{2} & -I \end{array}\right]<0. \end{array}$$

The Lyapunov function is chosen as $V=\delta^{T}X\delta$. The derivative of *V* is obtained as:

$$\begin{array}{cc} \dot{V}\left(t\right)= & {\dot{\delta }}^{T}X\delta +\delta^{T}X\dot{\delta }\\ = & \delta^{T}\left(H^{T}X+XH\right)\delta +\omega^{T}\varphi_{1}^{T}X\delta +\delta^{T}X\varphi_{1}\omega . \end{array}$$

With ω=0 and (12), $\dot{V}<0$ is satisfied; hence, the system (8) is asymptotically stable.

$$\begin{array}{cc} \; & \dot{V}+e^{T}e-\gamma^{2}\omega^{T}\omega \\ \; & =\left[\begin{array}{cc} \delta^{T} & \omega^{T} \end{array}\right]\left[\begin{array}{cc} H^{T}X+XH+P^{T}P & X\varphi_{1}+P^{T}\varphi_{2}\\ \varphi_{1}^{T}X+\varphi_{2}^{T}P & \varphi_{2}^{T}\varphi_{2}-\gamma^{2}I \end{array}\right]\left[\begin{array}{c} \delta \\ \omega \end{array}\right] \end{array}$$

By the Schur complement to (12), one obtains:

$$\begin{array}{c} \left[\begin{array}{cc} H^{T}X+XH+P^{T}P & X\varphi_{1}+P^{T}\varphi_{2}\\ \varphi_{1}^{T}X+\varphi_{2}^{T}P & \varphi_{2}^{T}\varphi_{2}-\gamma^{2}I \end{array}\right]<0. \end{array}$$

Therefore:

$$\begin{array}{cc} \; & \dot{V}<\gamma^{2}\omega^{T}\omega -e^{T}e,\\ \; & \int_{0}^{\infty }\dot{V}\left(\tau \right)d\tau <\int_{0}^{\infty }\gamma^{2}w^{T}\left(\tau \right)w\left(\tau \right)d\tau -\int_{0}^{\infty }e^{T}\left(\tau \right)e\left(\tau \right)d\tau . \end{array}$$

Under the zero initial condition, $V\left(\infty \right)<\gamma^{2}{∥w∥}^{2}-{∥e∥}^{2}$. Hence, the error system satisfies ${\left\|\;e\;\right\|}_{L_{2}}<\gamma \;{\left\|\;\omega \;\right\|}_{L_{2}}$ for ω≠0.

Inserting the solution of (6) into (12), Theorem 1 is obtained. Then, the theorem is proved. □

From Theorem 1, the prescribed level of noise γ determines the feasibility of (11). According to robust control theory, γ can be selected by the following optimization problems:

(13) $$\begin{array}{cc} \; & \min(\gamma )\\ \; & \;s.t.\;\;X=X^{T}>0,\\ \; & \;\;\Sigma <0. \end{array}$$

This optimization problem can be solved with the YALMIP toolbox [27].

The proof process of Theorem 1 embodies the following observer design steps:

1. Model the battery system as a descriptor system (2);
2. Determine the matrix Φ by ΦE=0;
3. Determine the matrix $N^{{}^{\prime }}$ by the (9);
4. Choose the prescribed level of noise γ by optimization problems (13);
5. Solve the feasible solution of (11) given by Theorem 1;
6. Calculate the matrices *H*, *J*, *P*, *Q*, *R*, $\varphi_{1}$, and $\varphi_{2}$;
7. Convert the virtual output into the actual measurable output by $\bar{y}=y-Du$.

From the above steps, there are some parameters that need to be chosen. γ determines the disturbance rejection level of the observer, which usually cannot be a large value. $N^{{}^{\prime }}$ only needs to satisfy (9), and the numerical size of each element in the matrix $N^{{}^{\prime }}$ has little effect on the final result. Therefore, compared with the existing method for SOC estimation, the proposed $H_{\infty }$ observer does not require complex tuning.

## 4. Results and Discussion

In order to illustrate the superiority of the proposed $H_{\infty }$ observer, this paper will compare it with the PI observer [7] and SMO [15]. To ensure the fairness of the test, the parameters of battery model (Figure 2) are shown in Table 1.

The piecewise algebraic relationship between $v_{oc}$ and SOC is $v_{oc}=1.2s+3$; bring the battery parameters and $v_{oc}$ functions into the model, and the battery modeling is complete.

It can be verified that the battery system whose parameters are shown in Table 1 satisfies Assumption 1; therefore, we can design an $H_{\infty }$ observer of the construction (4) by Theorem 1.

Take a non-zero solution of the equation ΦE=0 as $\Phi =\left[\begin{array}{ccc} 0 & 0 & 1 \end{array}\right]$. Note that $N^{{}^{\prime }}=\left[\begin{array}{ccc} 1 & 0 & 0\\ 0 & 1 & 1 \end{array}\right]$ satisfies (9). Based on the $\gamma_{min}=0.7124$ from the optimization problem (13), we take γ=1.1, use the YALMIP toolbox to solve the (11), and obtain the $H_{\infty }$ observer as:

(14) $$\begin{array}{cc} \dot{z}= & \left[\begin{array}{cc} -0.6694 & -0.5393\\ 0.3236 & -1.3969 \end{array}\right]z+\left[\begin{array}{cc} -0.0027 & -1.6735\\ -0.0003 & 0.8091 \end{array}\right]u+\left[\begin{array}{c} 1.0971\\ 1.1272 \end{array}\right]y,\\ \hat{x}= & \left[\begin{array}{cc} 0.9095 & -0.0754\\ -0.7095 & 0.9409\\ 0.8825 & -0.2646 \end{array}\right]z-\left[\begin{array}{cc} 0.0004 & 0.2262\\ 0.0003 & 0.1774\\ 0.0013 & -2.2062 \end{array}\right]u+\left[\begin{array}{c} 0.1508\\ 0.1182\\ 0.5292 \end{array}\right]y. \end{array}$$

As a comparison, the PI observer applied in the technique proposed in [7] is:

(15) $$\begin{array}{cc} \dot{\hat{x}}= & \left[\begin{array}{ccc} -0.0111 & 0.0111 & 0\\ -0.8333 & 0.8333 & 0\\ 0.8222 & 0 & -0.8222 \end{array}\right]\hat{x}+\left[\begin{array}{c} -0.0000324\\ -0.0025\\ 0.003322 \end{array}\right]u\\ \; & +\left[\begin{array}{c} 0.1845\\ 0.005\\ 0.2 \end{array}\right]\left(y-\hat{y}\right)+\left[\begin{array}{c} 0.1\\ 0.005\\ 0.2 \end{array}\right]\alpha ,\\ \dot{\alpha }= & 0.01(y-\hat{y}),\\ \hat{y}= & \left[\begin{array}{ccc} 0 & 0 & 1 \end{array}\right]\hat{x}, \end{array}$$

where $\hat{x}$ and $\hat{y}$ are the estimate of ${\left[\begin{array}{ccc} v_{oc}^{T} & v_{c}^{T} & v_{t}^{T} \end{array}\right]}^{T}$ and $v_{t}$, respectively. Notice that u=i.

The SMO proposed in [15] is shown as:

(16) $$\begin{array}{cc} \dot{\hat{x}}= & \left[\begin{array}{cc} -0.8333 & 0.8333\\ 0.00926 & -0.00926 \end{array}\right]\hat{x}+\left[\begin{array}{c} 0.0025\\ 0.000027 \end{array}\right]u-\left[\begin{array}{c} 1.667\\ 0.0185 \end{array}\right]\left(y-\hat{y}\right)+\left[\begin{array}{c} 0.0025\\ 0.000027 \end{array}\right]v,\\ v= & \left\{\begin{array}{cc} -\frac{661.376}{y-\hat{y}}\left(6.048\times 10^{-3}\left(y-\hat{y}\right)+6.929\times 10^{-5}{\left(y-\hat{y}\right)}^{1.4}\right) & y-\hat{y}\neq 0\\ 0 & y-\hat{y}=0 \end{array}\right.,\\ \hat{y}= & \left[\begin{array}{cc} 0.5 & 0.5 \end{array}\right]\hat{x}+0.0025u, \end{array}$$

where $\hat{x}$ and $\hat{y}$ are the estimatse of ${\left[\begin{array}{cc} v_{c}^{T} & v_{oc}^{T} \end{array}\right]}^{T}$ and $v_{t}$, respectively. The input *u* is the current *i*.

**Figure 3:**
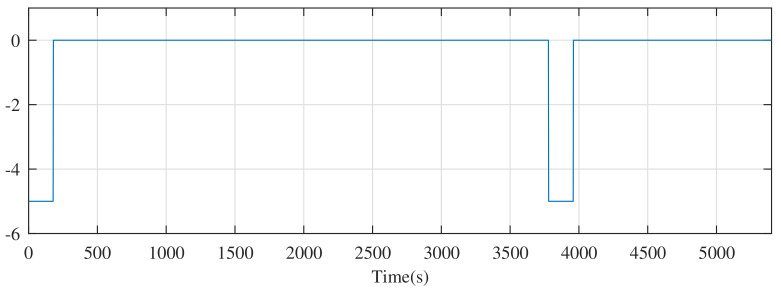
The current of the constant current discharge experiment.

**Figure 4:**
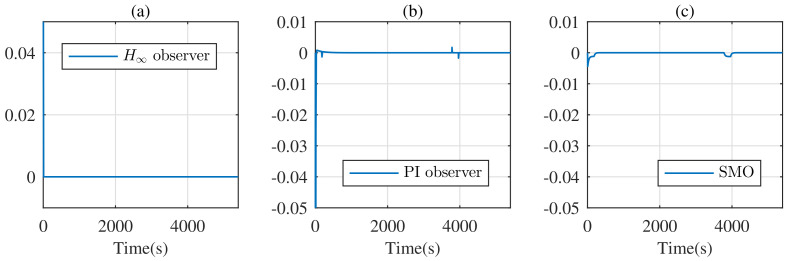
SOC estimate error under the constant current discharge experiment: (**a**) based on the $H_{\infty }$ observer; (**b**) based on the PI observer; (**c**) based on SMO.

The constant current discharge experiment, to evaluate the performance of the observer, is employed as follows: choose a discharge current of 5 A whose discharge period is 3980 s, and discharge for 180 s. Figure 3 shows the current of the constant current discharge experiment. For fairness, the known initial SOC of the battery model is 0.8, and Figure 4 shows the estimate errors from the different observers in this experiment.

From Figure 4, the $H_{\infty }$ observer and the PI observer can converge to zero quicker, with respect to the SMO. However, at each instant of discharge, the PI observer and SMO need a short period of adjustment to reach the steady state again, while the $H_{\infty }$ observer based on the descriptor system overcomes this drawback.

**Figure 5:**
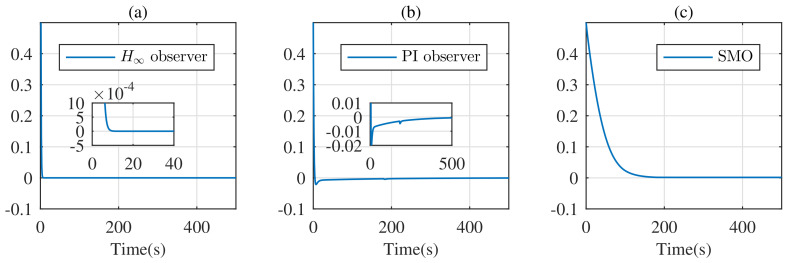
SOC estimate error under the constant current discharge experiment with inaccurate initial SOC: (**a**) based on the $H_{\infty }$ observer; (**b**) based on the PI observer; (**c**) based on SMO.

The initial conditions of the SOC are set as 0.3 for observers and 0.8 for the battery. In this case, the simulation result is shown in Figure 5. Due to the inaccurate initial SOC, there is large error of SOC from each observer at the initial moment. However, the estimate error of the $H_{\infty }$ observer converges to zero within 20 s, while the estimate error of the PI observer and SMO converge to zero within 500 s and 200 s, respectively. So, the $H_{\infty }$ observer is not sensitive to accurate initial SOC and has a fast convergence speed.

**Figure 6:**
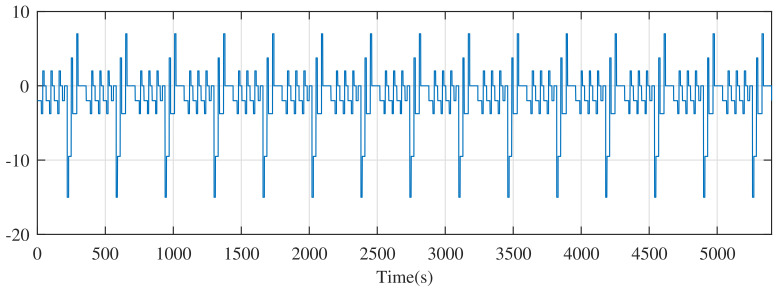
The current of the dynamic stress test.

The dynamic stress test (DST) is a standard test condition proposed by the Advanced Battery Association of the United States to simulate urban driving condition for electric vehicles. It is commonly applied to test the dynamic performance of SOC observers. The current of DST is shown in Figure 6.

**Figure 7:**
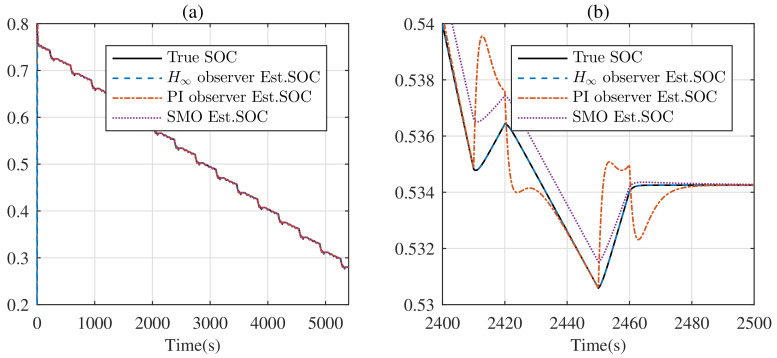
Real SOC and its estimate under the DST: (**a**) full graph; (**b**) zoomed graph.

Under the DST, the estimated SOC is shown in Figure 7. Generally, each observer can track the SOC. However, from Figure 7b, the true SOC is covered by the estimated SOC from the $H_{\infty }$ observer completely, which means the $H_{\infty }$ observer exhibits better performance for tracking the real SOC, with respect to the PI observer and SMO.

**Figure 8:**
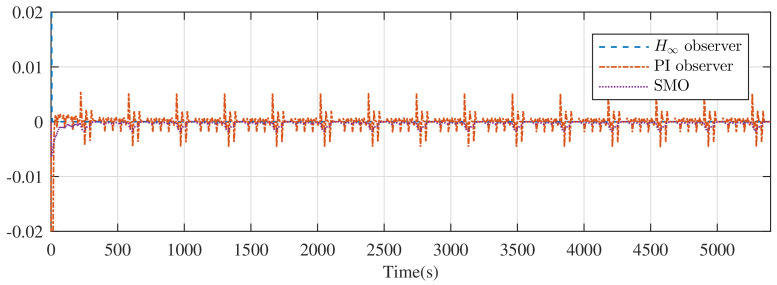
Error of SOC for DST from the $H_{\infty }$ observer, PI observer, and SMO.

The estimate error under the DST is plotted in Figure 8. Because of the reservation of the current dynamic performance, there does not exist pulse mode in the estimation error of the $H_{\infty }$ observer. Figure 7 and Figure 8 illustrate the outstanding dynamic performance.

**Figure 9:**
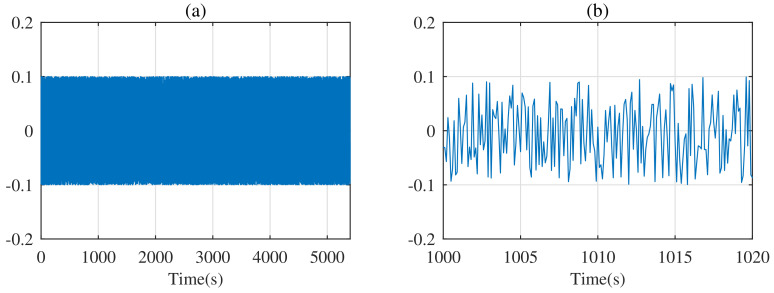
The uncertainty factor in the battery model: (**a**) full graph; (**b**) zoomed graph.

**Figure 10:**
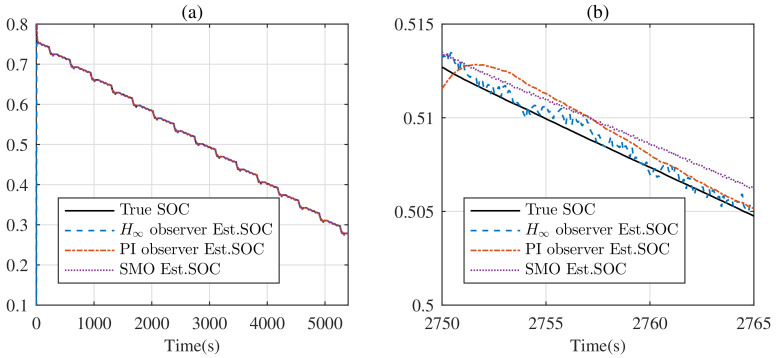
The real SOC and its estimate from the $H_{\infty }$ observer, PI observer, and SMO under parameter perturbations: (**a**) full graph; (**b**) zoomed graph.

We are also interested in measuring the performance of the proposed observer’s deleted structure (4) in the presence of parameter perturbations to see if there are improvements in the robustness, with respect to the PI observer and SMO. So, the case when the capacitance and resistance parameter perturbations are given (Figure 9) is considered. Figure 10 shows the behavior of the true SOC and its estimate when the uncertainty is present.

From Figure 10, due to the parameter perturbations, each observer is unable to track the true SOC accurately. However, the estimate error of SOC from the $H_{\infty }$ observer is less than the PI observer and SMO, which illustrates the robustness of the schema proposed in this paper.

## 5. Conclusions

To improve the accuracy of SOC observers and decrease the effects of the parameter perturbations, an $H_{\infty }$ observer, based on descriptor systems, is designed to estimate SOC. Firstly, the battery is modeled as a descriptor system without the extra derivative operation of nonlinear piecewise constraints. For the uncertainty, robustness nonlinear $H_{\infty }$ theory is employed to solve the observer. Furthermore, the design steps of a type of battery SOC observer are given systematically. The simulation results show that the $H_{\infty }$ observer based on descriptor systems is more effective, and the observation accuracy is higher with respect to the PI observer and SMO. The method proposed in this paper is not sensitive to battery parameter changes. Therefore, the proposed $H_{\infty }$ observer based on descriptor systems provides a new and effective online method to estimate SOC in REVs. The extension of our work to nonlinear $H_{\infty }$ observers is under study.

## Figures and Tables

**Table 1:** Parameters of the lithium battery.

$C_{N}$	$C_{C}$	$R_{e}$	$R_{c}$	$R_{t}$
18,000 F	200 F	0.003 Ω	0.003 Ω	0.001 Ω

## Abbreviations

The following abbreviations are used in this manuscript:

REVs	Renewable energy vehicles
SOC	State of charge
OCVM	Open-circuit voltage method
CCM	Coulomb counting method
KF	Kalman filter
SMO	Sliding-mode observer
PI	Proportional-integral
OCV	Open-circuit voltage
RC	Resistance–capacitance
LMI	Linear matrix inequality
DST	Dynamic stress test
RMSE	Root mean square error
MAE	Maximum absolute error
